# Nature connection, wellbeing and pro-environmental behaviour across an urban gradient: Understanding the regional sweet spot

**DOI:** 10.1007/s13280-025-02229-2

**Published:** 2025-08-31

**Authors:** Brenda B. Lin, Kate Sollis, Emily J. Flies, Pauline Marsh

**Affiliations:** 1 CSIRO, Land & Water Ecosystem Sciences Precinct, 41 Boggo Road, Dutton Park, QLD 4102 Australia; 2 Geography, Planning and Spatial Sciences, University of Tasmania, Sandy Bay Campus, Hobart, TAS 7001 Australia; 3 Wicking Dementia Research and Education Centre, University of Tasmania, Hobart, TAS 7001 Australia; 4 CSIRO Environment, Brisbane, Australia

**Keywords:** Conservation, Environmental behaviour, National analysis, Nature relatedness, Regional development

## Abstract

While there is growing understanding of the impact of urbanisation on nature access and nature connection, little is known about this pattern across the rural-urban gradient. Through a nationally-representative survey, we explore relationships of nature connection, wellbeing, and pro-environment behaviour across remote, regional, and urban Australia. We also examine two open-ended questions about an individual’s everyday interaction and meaningful experiences with nature. The results show non-linear relationships with stronger levels of nature connection in remote and outer regional areas of Australia, while personal well-being and pro-environmental behaviour scores were higher in outer regional Australia. Open-ended questions revealed that increasing urbanisation led to more limited interactions or meaningful experiences with nature, and individuals in remote and outer regional areas feel immersed in nature, while individuals in major cities travel to visit nature. Regional areas appear to provide opportunities for immersive nature while also access to key services that contribute to wellbeing.

**Supplementary Information:**

The online version contains supplementary material available at 10.1007/s13280-025-02229-2.

## Introduction

In the last decade, a large body of research on nature relatedness and nature connection has been undertaken within environmental psychology and socio-ecological systems to better understand an individual’s position and framing of themselves to nature (Ives et al. 2018; Wyles et al. 2019). This body of previous research has resulted in a number of concepts and frameworks regarding humans and the way they relate to nature, especially concerning the different ways that individuals can interact with or value nature (Díaz et al. 2015; Chan et al. 2016; Fish et al. 2016). Previous research has also sparked an interests in understanding how human-nature relationships and values differ across geographic regions and spatial scales (Díaz et al. 2015).

Much of the research around human-nature relationships has been concentrated in cities and in urban settings (Soga and Gaston 2016; Ives et al. 2017). This is largely due to the urban ecology work of the early 2000’s which led ecologists to be concerned about the rapid loss of biodiversity in cities, and thus the loss of opportunities to interact with the natural world (Pyle 2003; Miller 2005). This raised concerns with conservationists, who considered that the loss of opportunity would lead to an “extinction of experience”, that would result in further reduced awareness of and concern for the environment (Pyle 1993). The long-term consequences for human society from the loss of nature and biodiversity in cities through time are that each generation has less opportunity to experience nature. The theory is that this “ratcheting” down effect can lead to not just less understanding of nature, but also less valuing of its existence (Pyle 2003; Miller 2005). Here we are applying the broader definition of ‘nature’, inclusive not just of ‘wild’ areas but diverse domestic non-human species such as those found in gardens, backyards and urban green spaces (Soga and Gaston 2020). More recent work has shown that the loss of interaction with nature can reduce the positive emotions, attitudes, and behaviour one may feel regarding the environment, creating a cycle of disaffection toward nature (Soga and Gaston 2016).

There continues to be extensive interest in the concept of the extinction of experience, and how this trend can be reversed to bring back nature-interaction benefits to increasingly urban populations (Schuttler et al. 2018; Colléony et al. 2020; Oke et al. 2021). Affordance theory suggests that people can (re)learn about nature and how to interact with it in difference contexts and setting (Hadavi et al. 2015; Lennon et al. 2017), such that new, meaningfully interactions are developed as the environment changes (Marcus et al. 2016; Lin and Andersson 2023). Connecting people back to nature through citizen science experiments (Schuttler et al. 2018; Colléony et al. 2020), for example, or looking at other types of landscape management that allows for greater chances of connection (Colléony et al. 2017) may be important interventions in urban areas. This is especially necessary when thinking about children as early connections to nature can have life-long influences (Soga et al. 2020).

Like human-nature research, the primary focus of research on nature connection and wellbeing globally has been concentrated in larger capital cities (Dean et al. 2018; Uhlmann et al. 2018; Lin et al. 2023). Investigating and understanding nature relatedness and nature connection in regional and remote areas is important because we have less knowledge of how these different environments compare to the urban landscape. This type of comparative research is starting to be explored in the recent literature. In a study in Isreal, researchers found that people who currently live or used to live in rural areas during their childhood had higher scores of nature relatedness than their urban counterparts (Bashan et al. 2021). Other research in Ecuador and Spain showed that rural students had greater access to nature, spent more time in nature, and had a more positive view of nature in comparison to urban students (Macias-Zambrano et al. 2024). In a recent Australian study, self-described childhood living environment was a strong predictor of nature connectedness with people who grew up in environments described as ‘regional/remote’ having higher connection than those who stated that they grew up in a city (Sollis et al. 2025).

Australia is a pertinent case study to examine nature connection over urban gradients. Over the past 30–50 years, Australia has experienced a rapid transition toward greater urbanisation with a growth in coastal cities (Newton et al. 2017). Researchers cannot go back in time to examine how individuals used to interact with their environments, but we can study how nature relatedness and nature connection vary across rural to urban spectrums in contemporary Australia. We can do this by studying regional and rural towns, somewhat as a proxy—or analogue—of the size and structure of Australian cities at various historical points. Of course, it is important to recognise that there have been large changes through this period. Overtime, there has been varied growth and vibrancy of regional and rural towns of Australia, where much of the agriculture and resource economy stems from and remains (McManus et al. 2012; Buys et al. 2015)., Technology and lifestyles changes have led to differences that of course cannot be adjusted for in an analysis. However, we may use these remoteness gradients to increase our understanding of how changes in infrastructure and availability across the rural and urban transition may impact on an individuals’ interactions and experiences with nature.

In this present study, we investigate how nature connection, well-being, and pro-environmental behaviour differ based on the environmental context, specifically across the rural to urban gradient. We analyse survey responses from over 4000 participants, with strong representation across rural and regional areas, across Australia to investigate differences in self-reported nature connection, well-being, and pro-environmental behaviour. We analyse these relationships across four Australian Bureau of Statistics classifications of remoteness based on participant postcode (ABS July 2021–June 2026). We apply quantitative and qualitative analysis techniques to two survey questions that invited open-ended responses concerning everyday interactions and meaningful experiences of nature. We use this information to gain deeper insights into how differences in environments may impact on relationships of well-being, pro-environmental behaviour, and nature connection. Across these categorisations (very remote/remote, outer regional, inner regional, major metropolitan), we are able to gain a more nuanced understanding of how the experience of nature and its impact varies across urban gradients which can give us insights into the extinction of experience in Australia.

## Materials and methods

### Online survey design

A survey was designed to examine nature connection, personal well-being, and pro-environmental behaviour at a national scale across Australia. The survey was distributed nationally by recruiting participants through Online Research Unit’s existing panel sample. This project was approved by the University of Tasmania Human Research Ethics Committee (Project ID 28109). All participants fully consented to being involved in the survey.

A total of 4114 individuals took part in the online survey, with 108 responses excluded following a data cleaning process. The data cleaning process removed survey participants who had answered very quickly (completed the survey in less than 20% of the median completion time), straight-liners (those who gave identical responses across multiple questions), and individuals who provided nonsensical answers to open-ended questions (no survey participants were excluded based off this final criterion). Ultimately, 4006 de-identified individuals remained in the final sample, reflecting a 5% response rate typical of large online panels (Daikeler et al. 2020). We aimed to ensure that there was demographic diversity across multiple variables. The sampling strategy sought to obtain approximately equivalent samples sizes in each state and territory, and representativeness across demographics such as gender, age and education status. Table 1 provides detail on demographics of the sample.

**Table 1:** Detailed demographics of the survey participants within the study showing the representative proportions of each variable in each category

Variables		n	Percentage	National benchmark (2021 Census)
Age	18–30	832	20.77	21.6%
	31–50	1,289	32.18	35.0%
	51–70	1,334	33.3	29.2%
	> 70	551	13.75	14.3%
Gender	Male	1,913	47.84	49.3%
	Female	2,074	51.86	50.7%
	Non-binary	12	0.3	N/A
Identifies as Indigenous	Identifies as Aboriginal/Torres Strait Islander	79	1.99	3.4%
	Non-Indigenous	3,883	98.01	96.6%
Disability	Has disability	363	9.06	N/A
	No disability	3,643	90.94	N/A
Linguistic diversity	Speaks a language other than English at home	365	9.11	22.3%
	Speaks only English	3,641	90.89	77.7%
Educational status	Has not completed Year 12	353	8.81	8.1%
	Year 12 certificate	561	14	33.4%
	Certificate/Diploma	1,183	29.53	31.5%
	Undergraduate	1,052	26.26	19.6%
	Postgraduate	857	21.39	7.4%
Employment status	Full-time	1,716	42.86	38.2%
	Part-time	452	11.29	21.3%
	Casually employed	243	6.07	N/A
	Self-employed	258	6.44	N/A
	Home duties/volunteer work	208	5.19	N/A
	Retired	929	23.2	N/A
	Not working or studying	133	3.32	N/A
	Student only	65	1.62	N/A
Income quintile	Lowest income quintile	573	16.17	20%
	Second-lowest income quintile	813	22.94	20%
	Middle income quintile	527	14.87	20%
	Second-highest income quintile	1,015	28.64	20%
	Highest income quintile	616	17.38	20%
Current State	NSW	537	13.4	31.8%
	Vic	533	13.31	25.6%
	Qld	534	13.33	20.3%
	SA	534	13.33	7.0%
	WA	535	13.35	10.5%
	Tas	520	12.98	2.2%
	ACT	507	12.66	1.8%
	NT	306	7.64	0.9%
Remoteness Category	Very Remote/Remote Australia	152	4	2
	Outer Regional Australia	720	18	8
	Inner Regional Australia	979	24	18
	Major Cities of Australia	2155	54	72

The population of Australia is not evenly distributed and concentrated in a few major cities. In this study, we adopt the remoteness categorisation created by the Australian Bureau of Statistics, which divides Australia into five classes of remoteness, characterised by a measure of relative geographic access to services. The five classes are: Very Remote Australia, Remote Australia, Outer Regional Australia, Inner Regional Australia, and Major Cities of Australia. These categories are highly spatially distributed with major cities on the coast and areas becoming more remote in more central and northern areas of Australia. The categorisations change over time, but the categorisation used in the analysis within this paper are based on the 2021 Census data (ABS July 2021–June 2026). As of June 30 2023, *Major cities* are home to 72% of the total population, 18% live in *Inner regional* Australia*, 8% live in Outer regional* Australia, with the remainder (2%) living in *Remote and very remote* areas (Australian Institute of Health and Welfare 2024).

While these categorisations have been calculated to represent relative geographic access to services, the categories also use road distance and population as a proxy measure for service availability (ABS July 2021–June 2026). This is turn may provide an indication of the level of infrastructure development in each category and corresponding availability and access to different types of nature. For example, in the Major Cities category, developed urban areas may primarily have parks and other municipal green spaces that are planned and manicured with small pockets of remnant vegetation. Inner Regional areas may represent coastal satellite cities close to Major Cities, and while there are parks, there may be beach and mountains nearby that allow for additional nature experiences. Outer Regional areas may be further inland or along the coast further away from Major Cities. Remote and Very Remote areas represent a large proportion of the continent with nature represented by coast, forest, desert, and resource industries such as agriculture and mining. For a map of the categories visually presented spatially across Australia, please refer to the Australian Bureau of Statistics Remoteness Structure page (ABS July 2021–June 2026)^1^.

Despite the majority of Australians living in major cities and inner/outer regional areas, we set state-based sample quotas of: 50% capital cities, 25% major cities outside capitals, 25% other regions so as to gain sufficient data to analyse across this gradient. For the analysis, the survey participants from very remote and remote categorisations were combined because of the low participant number for each of these categories; however, this spread is representative of the population proportion within these categories with the representation across states and territories providing more evenness of sampling. This led to 152 responses in the very remote/remote classification, 720 in Outer Regional Australia, 979 in Inner Regional Australia, and 2155 in Major Cities of Australia (Table 1).The sampling strategy also aimed for quotas across states and territories (approximately 500 per region) though the quota for the Northern Territory (306 participants) was not fully met, consistent with its historical survey participation challenges (Australian Bureau of Statistics 2022). Within-state quotas were set for education levels and urban and rural areas to ensure representation. Overall, the sample displayed balanced representation across key demographics such as gender, age, education, and income (Table 1, but also see (Sollis et al. 2025).

Several scales were also used in the survey to better understand a respondent’s nature connection, personal well-being, and pro-environmental behaviour (PEB) orientations. All of these scales, except the PEB, have been psychometrically validated and are commonly used in research surveys. An analysis of internal consistency reliability (Cronbach’s alpha) was calculated for each scale. The coefficient alpha is reported for each scale within the description of the scale construct. A full copy of the survey has been published and can be found in Sollis et al. (2025). A more detailed overview of the three main constructs can be found below:

- **Nature connection**: Nature connection was measured using the CN-12 which comprises a series of 12 questions regarding nature connection across three dimensions (identity, experience, and philosophy) (Hatty et al. 2020). For example, an identity domain question asked “My relationship to nature is a big part of how I think about myself”, an experience domain question asked “I like to get outdoors whenever I get the chance”, and a philosophy domain question asked “Human wellbeing depends upon living in harmony with nature”. For each question, survey participants were asked to rate themselves on a scale from 1 to 7: from weak (1), to moderate (5), to strong (7). The 12 data items were averaged to calculate an overall score as described by Hatty et al. (Hatty et al. 2020), with the data items in each of the dimensions averaged to obtain the dimension scores. The CN-12 was identified as the most appropriate primary variable for measuring nature connectedness due to it being developed and validated within the Australian context. The Cronbach alpha coefficient for this scale was 0.95, showing a high level of internal consistency.
- **Well-being**: Well-being measures were based on a scale developed within the Australian context: the Personal Wellbeing Index (PWI). The PWI is widely used, both within Australia and internationally, and measures life satisfaction in seven domains of life: standard of living, health, achieving in life, personal relationships, safety, sense of community, and future security (International Wellbeing Group 2013). In the survey, the question asks “Thinking about your own life and personal circumstances, how satisfied are you with the following aspects of your life:” and asks specifically about your life as a whole, your standard of living, your health, what you are achieving in life, your personal relationships, how safe you feel, feeling part of your community, your future security, and the quality of your local environment. Survey participants were asked to rate each aspect from zero to 10, with zero representing no satisfaction as all to 10 representing completely satisfied. The scores for the sub-questions were averaged for analysis. The Cronbach alpha coefficient for this scale was 0.89, showing a high level of internal consistency.
- **Pro-environmental behaviour**: Pro-environmental behaviour was measured by adapting the pro-environmental advocacy behaviours scale from the Victorians Valuing Nature survey (Squires et al. 2022), a scale that has been implemented and test in the Australian context. In the survey, a question “How often do you engage in the following environmental behaviours?” was presented, followed by a series of questions regarding one’s behaviour such as “I bring up environmental issues in conversation with my peers” or “I sign petitions about environmental issues I’m concerned about”. Nine of the options were part of the original survey, and an additional item, ‘I vote for people, parties, or policies that support nature’ was included in our analysis to further enhance the measure. Survey participants were asked to gauge their level of behaviour by rating their response as (1) Never, (2) Rarely, (3) Sometimes, (4) Often, (5) Very often, NA (Not Applicable). The scores of the sub-questions were averaged for the analysis. The Cronbach alpha coefficient for this scale was 0.93, showing a high level of internal consistency.

### Open ended questions

Two open ended questions from the survey were included in this analysis. These questions asked about respondent interactions with nature as well as the meaningful experiences that they have had with nature. Using open-ended questions enables participants to provide information that is not restricted by supplied options, and that allows us to gather qualitative data that will help answer the research question (Creswell and Clark 2017). To this end, the questions were:

- **Question 1: Everyday interactions with nature**: What kind of interactions do you have with nature in your everyday life? Please describe in your own words
- **Question 2: Meaningful experiences with nature**: Think of a meaningful experience you have had that shaped the way you think about ‘nature’? Please describe it in 1–3 sentences in your own words.

The responses provided by the participants to these questions were generally short phrases or a few short sentences.

### Analysis

Across the four categories of remoteness, we analysed differences in composite nature connection, personal wellbeing, and pro-environmental behaviour scores using a one-way ANOVA (Okoye and Hosseini 2024) to determine if there was a difference across the remoteness categories. This analysis was conducted in R using the *aov* function and a Tukey Honest Significant Differences test to test significant differences between groups using the *TukeyHSD* function (R Core Team 2015). The Tukey Honest Significant Differences (HSD) test was chosen because it can perform multiple comparisons to determine which specific pairs of means are significantly different from each other, while controlling the overall family-wise error rate (of making one or more Type I errors). It can be used to compare more than two groups and to understand the relationships between all pairs of groups. By comparing all possible pairs of means, Tukey’s HSD identifies which specific means are significantly different, providing more granular insights than a simple ANOVA alone. The detailed reporting of the each pairwise analysis in included in the supplementary materials (Table S1).

Open-ended responses to the questions on nature interactions and meaningful experiences were analysed using a hybrid inductive + deductive approach (Proudfoot 2023). Firstly, an inductive coded content analysis was conducted across all the responses to each question by one researcher [BL]. The number of codes according to each remoteness category were then counted. These numbers were then scaled by the total number of responses in the category to ascertain the percentage of responses per category. This initial analysis provided us a general understanding of the patterns of responses that were emerging from the two open-ended questions, and a visual understanding of frequency of responses and how they varied across the remoteness categories. Figures of these responses can be found in supplementary materials (Figs. S1 and S2).

Secondly, the codes were then synthesised by the authorship team [BL, KS, EF, PM] through a secondary process of reflexive thematic analysis (Terry and Hayfield 2020). This iterative process involved reflection, discussion and re-categorisation, and led to the generation of themes and subthemes across the overarching categories of nature interaction and meaningful experience (Table 2 in Findings). At this point, coding of “no interaction or limited interaction/experience” were (re)allocated to a category of “No interactions or experiences”; however, individuals who decided to not respond to one or the other question were allocated as “no response”. These individuals were allocated into a separate category and not included with the group that stated that they had “No interactions or experiences”. Subthemes were then further categorised according to the remoteness categorisation of the survey participants, and categorised according to broad indications of frequency of mentions of occurrence across the regions compared to one another—high, medium, low and rare (Tables 3 and 4 in Findings, based on raw data compiled and presented in Supplementary Fig. S1 (Interactions subthemes) and S2 (Experiences subthemes)).

**Table 2:** Themes and subthemes generated by analysis of open-ended responses

Themes	Subthemes (by question)	
	Everyday Interactions with Nature	Meaningful Experiences with Nature
Practical everyday activities	Exercise, walking	Exercise, walking
	Outdoor gardening	Gardening, taking care of plants
	Indoor gardening	Enjoying healthy foods
	Working outside	Working outside
Being outside	Enjoying and observing nature	Enjoying/respecting the beauty of nature, time in nature
	Being outside in nature	Listening to sounds of nature
	Fresh air	Fresh air
	Watching and interacting with animals	Watching and interacting with animals
	Surrounded by nature, living in nature	Living around nature (coastal and rural landscapes)
		Holidaying in nature (camping, traveling to nature locations)
		COVID Freedom
People	Spending time outside with kids, family, friends	Spending time outside with kids, family, friends
	Being away from people	Childhood experiences
		Aboriginal, cultural experiences
Conservation	Taking care of nature, conservation	Taking care of nature, conservation
Emotional, Spiritual	Spiritual and emotional connection to nature	Feeling immersed in nature at a spiritual level

We explored the four different remoteness groups to understand the commonalities and differences between groups (Table 3), and to understand how these may explain differences in nature connection, personal wellbeing, and pro-environmental behaviour scores.

In the results and findings section that follows, we firstly present the results of the ANOVA analysis, and then the thematic analysis findings by question.

## Results

### Analysis of nature connection, personal wellbeing and pro-environmental scores

**Fig. 1:**
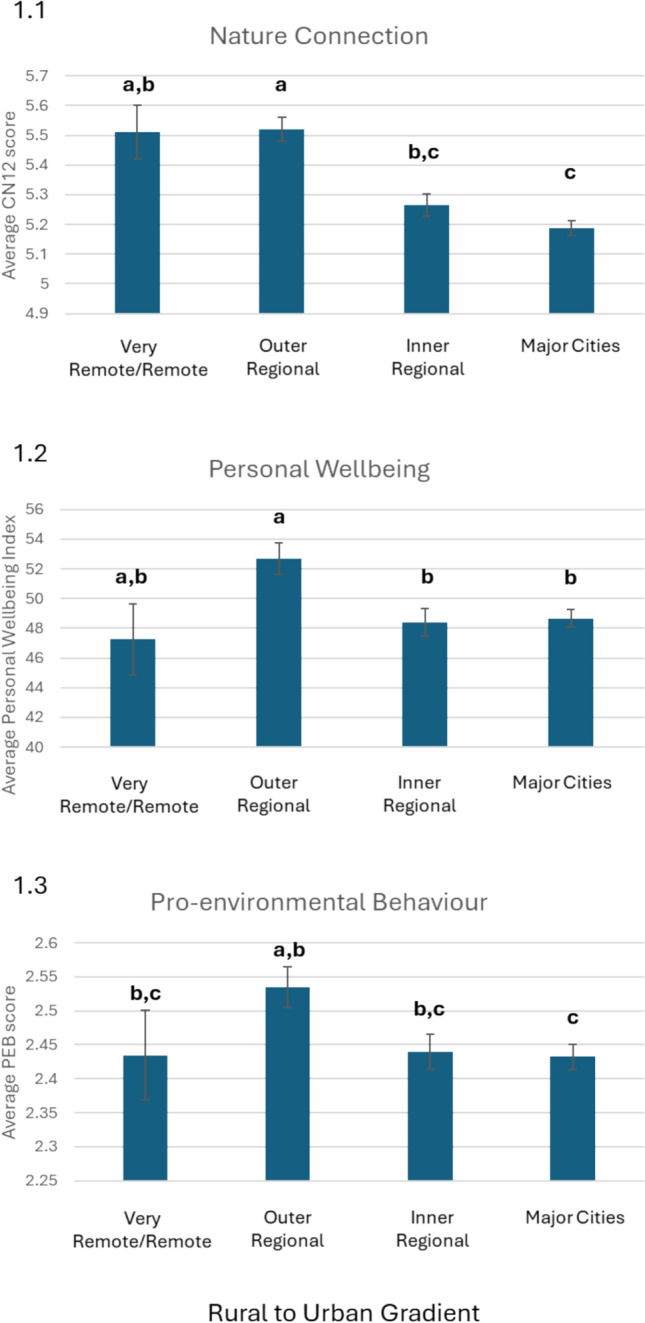
Results of a one-factor Anova analysis comparing the nature connection (1.1), personal wellbeing (1.2), and pro-environmental behaviour (1.3) scores across the rural to urban continuum based on the remoteness categories from the Australian Bureau of Statistics. Anova models were significant, and significant differences between group were determined with a Tukey Honest Significant Difference tests. Error bars represent the standard error. Differences in lettering (a versus b) indicate a significant difference between the categories. Detailed statistical analyses can be found in Table S1

The results from the analysis of nature connection, personal wellbeing, and pro-environmental behaviours showed significant differences in the remoteness categories. For nature connection (CN-12 scale), the results showed that Outer Regional (mean = 5.52, SD = 1.10) had a significantly higher average level of nature connection than the individuals from Inner Regional areas (mean = 5.26, SD = 1.16) and Major Cities (Mean = 5.19, SD = 1.18), although there was some overlap with Very Remote/Remote (mean = 5.51, SD = 1.11) participants (Fig. 1a) (Anova: *p* < 0.000; F = 17.04; Tukey HSD test results in Table S1) whose nature connection was still higher than for people in Major Cities.

This pattern is similar to the results for personal wellbeing (PWI scale), where the Outer Regional participants (mean = 52.7, SD = 28.48) had significantly higher personal wellbeing than the Inner Regional and Major Cities, but some overlap with the Very Remote/Remote category (Fig. 1b, *p* = 0.005, F = 4.26, Tukey HSD results in Table S1). The results for the pro-environmental behaviour measurement was less clear; however, in this case the Outer Regional participants were also the group that scored the highest although only significantly different from the Major Cities group (Fig. 1c , *p* = 0.03, F = 2.91).

### Findings from the open-ended questions

Themes and subthemes for both open-ended questions (everyday interactions with nature and meaningful experience with nature) are presented in Table 2. These results have broad thematic categories of practical everyday activities, being outside, people, conservation, and emotional/spiritual reasons. Some subthemes, such as “exercise, walking” were present in both questions, while there were some subthemes unique to each question such as “COVID Freedom” or “Aboriginal or Cultural Experiences” in the second question.

#### Question 1: Everyday interactions with nature

There were a number of common subthemes that appeared across the remoteness categories regarding interactions with nature. A common subtheme was that people interacted with nature by walking and exercising in nature. This included a wide range of activities such as walking with their dogs in nature, hiking, cycling, boating, horseback riding, swimming, and surfing, in addition to other recreational activities that can be done outside (Table 3, Table S3 in Supplementary Information for quotes). This subtheme occurred across all remoteness categories.

**Table 3:** Frequency of occurrence of major subthemes for ‘interaction with nature’ that were generated by analysis of responses from the rural to urban gradients. Further description is included in the Findings

Interactions with Nature				
	Very remote and remote	Outer regional	Inner Regional	Major city
*Subthemes*				
Exercising, walking	High	High	High	High
Enjoying and observing nature	Medium	Medium	Medium	Medium
Gardening outdoors	Medium	Medium	Medium	Medium
Watching and interacting with animals	Medium	Medium	Medium	Medium–Low
Surrounded by nature, living in nature	Medium	Medium	Medium	Rare
Being outside in nature	High	Med-High	High	Medium
Working outside	Medium	Low	Low	Rare

Another common subtheme across the remoteness categories was enjoying and observing nature outdoors as well as gardening outdoors. These subthemes were all provided at a high frequency, with a high proportion of participants in each groups stating these as the main ways that they interact with nature on a daily basis. The subthemes of watching and interacting with nature and being outside in nature were also commonly stated, but at a lower frequency and with some differences between remoteness categories.

Certain subthemes demonstrated greater differences between groups. For example, the subthemes of being surrounded by nature or living in nature and working in nature, were more likely expressed in more remote and rural areas, compared to the major cities (see Table 3). A more detailed overview of the distinguishing characteristics between the groups around spiritual and emotional connections to nature, conservation behaviour, and other subthemes follows. Nuanced differences across the regions are highlighted with exemplary quotes provided in Table 4 (separate document).*Very Remote/Remote*: Individuals in this category frequently described enjoying and observing the landscape and the scenic views of nature as their everyday interaction with nature. They often mention that enjoy being able to watch and interact with animals when they are outside. Many responses in this category also included mentions of being outside in both their general living situation (“surrounded by nature”) as well as their chosen daily lifestyle. This includes many participants in this category expressing that they spend considerable time outside for recreation and relaxation, but their time at work may be outdoors as well.

*Outer regional*: Individuals in Outer Regional areas of Australia stated they interacted with nature because they were surrounded by it and lived in it, mentioning they had easy and immediate access to interact with nature. People noted that they were interacting with nature because it was part of their everyday lived experience, and they had learned to notice and appreciate the beauty and vitality of nature through these interactions. People in Outer Regional areas also indicated that they interacted with nature through a spiritual-emotional connections with the earth or Mother Nature. Many noted that conservation activities outdoors and spending time with children in nature was also important ways of interacting with nature.

*Inner regional*: Participants within the Inner Regional group also noted that they spend a lot of time being outside and visiting nature. In this group, like the two other groups above, people noted that they are surrounded by nature and they live in it. However, the descriptions of the types of nature are more variable—people mention living in rural, semi-rural, and urban fringe environments. In this group, people noted that they spend a lot of time in their yard and felt this space to be an important area for interacting with and enjoying nature. Some people specifically mentioned they saw nature through a window (at home or work) or while driving in their everyday life. In this group, some people noted that they work outside, but there were fewer mentions of interacting with nature with the purpose of conservation or to spend time with kids outside.

**Fig. 2:**
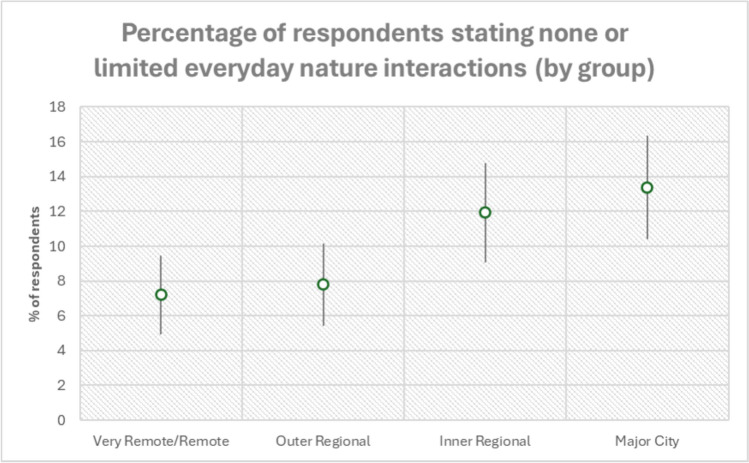
The percentage of people within each group that said they had none or limited everyday interactions with nature. Those who did not respond were not included in this number. The percentage is represented by the circle with 95% confidence intervals for population proportion, upper and lower limits represented by the line. The proportion of participants increases with the urbanisation gradient

*Major cities*: Major Cities participants rarely mentioned being surrounded by nature or that they lived in nature, unlike the participants in the other three categories. There were also fewer mentions of working outside as their primary way to interact with nature. However, many participants emphasised that their main way of interacting with nature was walking and exercising in nature—in their neighbourhood, through parks and in nature areas in the city. Many people in this group, like the Inner Regional group, described viewing or seeing nature from their home, decks, or balconies or from a car while driving to work or errands. Interacting with urban garden plants and animal in yards were regularly mentioned. Many people, however, said that they had minimal or limited experiences with nature or of being outside, either because they had limited time or disliked the outside world. Moreover, participants in this category had uncomfortable, even adversarial, relationships with nature. Major Cities participants described fewer everyday interactions with nature with the proportion of individuals who indicated limited or no interaction with nature increasing with the urbanisation gradient (Fig. 2).

#### Question 2: Meaningful experiences with nature

Common subthemes found across the four groups pertaining to meaningful experiences with nature were expansive (Table 4, Table S4 in Supplementary Information for quotes). First, as a cross-over with nature interaction findings, many people viewed their time walking and exercising in nature as a meaningful experience. Many mentioned walking or hiking in beautiful places and how this time spent walking led to meaningful experiences.

**Table 4:** Frequency of occurrence of major subthemes for categories ‘meaningful experiences with nature’ that were generated by analysis of responses from the rural to urban gradients. Further description is included in the Findings

Meaningful Experiences with Nature				
	Very remote and remote	Outer regional	Inner Regional	Major city
*Subthemes*				
Exercise, walking	Medium	Medium	Medium	Medium
Holidaying in nature (camping, traveling to nature locations)	High	High	High	High
Childhood experiences	Medium	Medium	Medium	Medium
Enjoying respecting the beauty of nature, time in nature	High	High	High	Medium
Watching and interacting with animals	Medium	Medium	Med-High	Medium
Living around nature (coastal and rural landscapes)	Medium	Medium	Med-High	Low
Feeling immersed in nature at a spiritual level	High	Medium	Medium	Medium

Many participants across groups also expressed that their meaningful experiences with nature were tied to a time when they were in nature and were awed by it, conjuring a feeling of respect for nature. Many people also said that the beauty of nature itself was a meaningful experience. This is related to opportunities to observe a beautiful landscape and watch wildlife in nature.

Another common subtheme for meaningful experiences were holiday/vacation experiences where people had opportunities to feel immersed in and to learn about the nature. This often involved memories of camping in nature, being in a space away from other people, or gaining opportunities to see other types of nature that may be different from their everyday nature. Meaningful experiences as children, and experiencing nature with family, were also often recalled by participants.

At a lesser frequency, participants mentioned that they had meaningful experiences by living near nature, in rural, coastal, and farming areas, and watching or interacting with animals. Living around nature allowed those in more remote or rural regions to feel immersed in nature in the areas of around them, while participants from Major Cities travelled to feel spiritually connected to nature.

A more detailed overview of findings by group for this question are presented below and the nuanced different and distinguishing characteristics and exemplary quotes shown in Table S3 in Supplementary Information.

*Very Remote/Remote*: Many people in this category felt that they had meaningful experiences when they were immersed in nature, more so than the other groups. Meaningful experiences were associated with situations that allowed people in this group to be in nature and experience the enormity and magnificence of nature. Many spoke about immersive experiences that allowed them to be away from other people or technology. This group included mentions of meaningful cultural experiences, and especially experiences with and on Traditional Owner lands, to experience the nature connections with the land.

*Outer regional*: Like the participants in the Very Remote/Remote groups, participants in Outer Regionals areas noted living in natural areas, being surrounded by nature, and also working outside in nature as their meaningful experiences. Many people noted the sensory aspects of nature—the smells, sights, and sounds that they felt—which allowed them to experience nature deeply. A meaningful experience that people had with nature sometimes involved an experience where they were taking care of nature or taking on a conservation action. There were many mentions of experiential learning with Aboriginal Australians or people from other First Nations cultures.

*Inner regional*: Inner Regional individuals noted meaningful experiences with nature based on watching animals in nature, more so than in other groups, with very specific experiences watching specific species. Many noted that they lived in an area with lots of nature, such as by the beach and the coast. There were frequent examples of participants associating nature with God and creation of nature as part of a spiritual experience. Although less frequently than for other groups, some people in this group wrote about a meaningful connection related to their own Aboriginality.

**Fig. 3:**
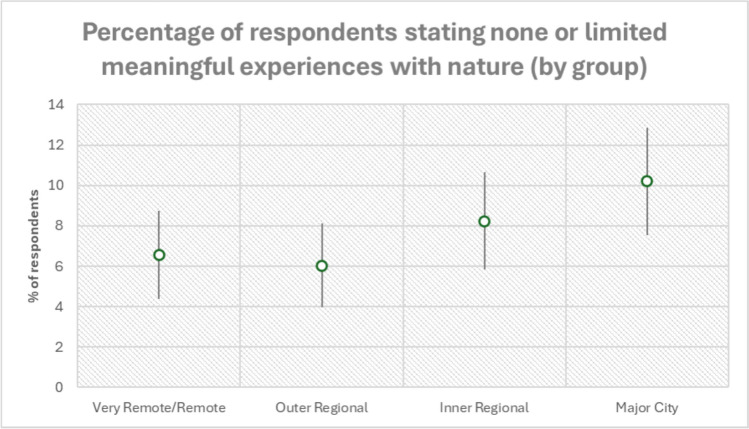
The percentage of people within each group that said that they had none or limited meaningful experiences with nature. This does not include individuals that did not provide any response. The percentage is represented by the circle with 95% confidence intervals for population proportion, upper and lower limits represented by the line. The proportion of participants increases with the urbanisation gradient, but less uni-directional than in the interactions with nature question

*Major cities*: There was an increase in people indicating they had no meaningful experiences as participants became increasingly more urban (Fig. 3). Participants from Major Cities responded more frequently that they had no meaningful experiences with nature or could not recall a time when it made an impression on them. Some people in this group did not see nature as an entity that they recognised. Some participants noted when nature helped them get through a hard time, while other saw meaning in nature as a sign of God. In this group, there was also the mention of how Covid-19 impacted their experiences with nature. This was especially around Covid Freedom and being able to get out into nature to leave their house when in lock down. There were a few instances of individuals saying that their meaningful experience with nature was based on Aboriginal and cultural nature experiences, but these took a more distant/philosophical view rather than an experiential one, observing that that they knew Indigenous communities to have a balanced relationship with nature.

## Discussion

Our results indicate that living in increasingly urbanised environments may be affecting human-nature relationships, wellbeing and pro-environmental behaviours. Analysis of open-ended responses regarding *how* people interact with and find meaning in nature revealed nuances and possible explanations for the differences within and between the groups. Because there is not readily available longitudinal data on these three factors, the space-for-time substitution of rural to urban gradient suggests that urbanisation may be contributing to an extinction of experience as people, and especially Australians, increasingly live in more urbanised settings.

### Differences in nature connection, personal wellbeing, and pro-environmental behaviour across the remoteness categories

The results show that the environmental context where an individual lives is highly relevant to one’s nature connection, personal wellbeing, and pro-environmental behaviour. First, people in Very Remote/Remote and Outer Regional areas of Australia have the highest levels of nature connection. Many of the participants from these geographies note that they are surrounded by nature and therefore feel that nature is a large part of their lifestyle. Many participants from these areas also note that they work outside, and therefore interacting with and experiencing nature is a big part of their daily life, not just in their leisurely or weekend activities. Previous work from the United States and Isreal (Skinner 2019; Bashan et al. 2021) has shown that there are different levels of nature connection between rural and urban population with the rural populations spending more time in nature.

Those living in Outer Regional areas of Australia score significantly higher in personal wellbeing and pro-environmental behaviours compared to people in Major Cities. However, the drop in wellbeing for people in Very Remote/Remote areas, though not significantly different than Outer Regional, suggests that the Outer Regional areas provide a level of access to both amenities and nature that supports high personal wellbeing as well as a desire to protect the environment. It is important not to conflate these two groups as both simply being rural. The remoteness categories developed by the Australian Bureau of Statistics explicitly use access to services to classify remoteness, therefore Very Remote/Remote locations have significantly poorer access to services than Outer Regional areas although both groups experience significantly less built environment in their immediate surroundings than more urban areas. It is also important to remember that despite the differences between Very Remote and Remote categories, they were combined in this analysis because of the small sample size from each of these groups.

Compared with Outer Regional, participants from Inner Regional and Major Cities areas had lower nature connection, personal wellbeing, and pro-environmental behaviour scores. These results align with research from Mexico which shows that rural children have a higher connection to nature and behave in a more pro-environmental way than urban children (Duron-Ramos et al. 2020), while in a study from Canada, researchers showed that individuals from urban and rural areas significantly differed in their levels of pro-environmental behaviour, but not in their levels of emotional connectedness to nature (Anderson and Krettenauer 2021). Another study from Eastern Europe showed that levels of remoteness and village size had an impact on an individuals’ attitudes toward pro-environmental behaviours and ability to act (Dąbrowski et al. 2022).

Although individuals living in Major Cities have less nature in the immediate surrounding environment, many participants still took the opportunity to leave the city to see nature or to seek out special places in the city to visit nature. Such a finding highlights the need to protect and conserve nature. Although urban dwellers can visit aspects of urban nature such as parks and remnant vegetation, their immersive experiences of nature are often in rural areas and not within the major cities. Many of the participants mentioned that their meaningful experiences were from visiting special national parks around the world and within Australia, which corresponds with tourism research showing that memorable experiences from nature tourism can lead to positive views of nature, and increased nature connection and pro-environmental behaviours (Chen et al. 2023). Thus, national conservation policies must consider the importance of national parks and immersive nature spaces in regional and rural areas and how the accessibility of such spaces impacts on the nature connection, personal wellbeing, and pro-environmental behaviour of the over 70% of Australians that live in major cities.

### The dichotomy of remote/very remote areas and wellbeing outcomes

Although both Very Remote/Remote and Outer Regional areas have high nature connection, the Very Remote/Remote areas do not have a significantly higher personal wellbeing or pro-environmental behaviour score even when compared with Major Cities. This is somewhat surprising as other research has shown that personal wellbeing and pro-environmental behaviour are both highly related to high levels of nature connection (Rosa et al. 2018; Whitburn et al. 2020; Grabowska-Chenczke et al. 2022). Many participants in the Very Remote/Remote categories mention that they spend a great deal of time outside and they are able to see nature readily from their homes. This accessibility to be in nature and to observe nature provides many opportunities to interact with nature and develop meaningful experiences. Thus, one would expect that the well-being and pro-environmental scores would follow the same pattern as the nature connection score. However, the rural/remote context of Australia is important to consider here. Although there has been an increased migration to regional and coastal areas from urban areas in recent years, due to a desire for lifestyle change and more affordable housing (Gurran et al. 2016; Guaralda et al. 2020), over 80% of Australians still live within cities less than 50 km of the coast (DCCEEW 2021). Thus, the population is largely urban and coastal. In remote areas there is less infrastructure in terms of towns and almost no access to services, including health services. The greatest health burden of disease has long been borne by regional and remote areas of Australia, and many health inequities exist on geographical lines; recent evidence suggests this trend is increasing (Flavel et al. 2024).

Australia has a large resource-based economy including extractive industries such as mining and agricultural production, representing almost 60% of the country’s exports (Reserve Bank of Australia 2019). Many of these jobs and locations for work are based in regional and remote towns where there are significant natural resources, but with very little infrastructure. Some remote areas are supported primarily by the employer, such as mining companies, provide temporary accommodation and food for the duration that these individuals are working. These individuals return back to Major Cities during their time off, such that they spend one to two weeks in a remote location and then one to two weeks in a major city, leading to transient populations (Haslam McKenzie 2010). This type of arrangement is termed as Fly-In,-Fly-Out or FIFO in Australia and impacts the health and stability of communities, families, and workers (Langdon et al. 2016), yet these FIFO jobs represent 17% of employment in regional areas (Asare et al. 2022). As some Very Remote/Remote participants in this study explained, they were only in these areas due to a type of job, and they were only there temporarily in order to complete a sometimes quite environmentally unfriendly job, such as mining. Other research has shown that this destruction of nature can have negative mental health impacts on these individuals (Perry and Rowe 2015; Gardner et al. 2018), which, combined with the poor access to services, may be related to the lower wellbeing we found in those areas.

Thus, the extinction of experience with nature and associated decreased wellbeing may also be occurring in remote areas, especially for those that work in extractive industries such as mining. It is also important to consider that in Remote areas, Australian agriculture is dominated by extremely large agricultural systems, often run in intensive or monocultural settings with little support for biodiversity. This lack of biodiversity and nature in remote settings suggests that we cannot expect residents of Remote and Very Remote areas to necessarily have access to nature or nature interactions that support connection, wellbeing and pro-environmental behaviours, and that this access may in fact be higher in Outer Regional areas.

### Childhood experiences and social experiences in nature can have long-term impacts

Across all regions, there was a clear relationship between the development of nature connection through social and intergenerational experiences with friends and family, in nature spaces. This included activities such as camping in nature, hiking or walking, and gardening. Many people found that the time that they spent with their families in nature taught them how to value and respect nature, how to protect nature and be part of nature. Such significant moments in childhood can impact on one’s connection to nature throughout life and the wellbeing benefits they gain from these experiences (Richardson et al. 2021), and national conservation policy and planning must consider how to bring more children in urban areas into nature spaces in order to provide immersive experiences more equally, but also to develop a new generation of individuals who value and promote the protection of the environment.

Many also mention that camping with their families when they were young was an important experience for them. Outdoor recreation and nature tourism can impact on individuals understanding of nature and lead to greater sustainability behaviour (Winter et al. 2019; Alcock et al. 2020). This is also supported by research which has shown that people who participated in a high diversity of outdoor activities as children, and people who participated in more outdoor activities as adults, also showed a stronger connection to nature (Skinner 2019). For example, longitudinal studies of young people in rural upstate New York found that participants who spent more time playing outdoors at age 6 reported more pro-environmental behaviours at age 18 (Evans et al. 2018). Thus, high nature connection can still be achieved or maintained in more urbanised settings if there are opportunities to visit quality nature spaces that leave memorable experiences, or if people have experiences from childhood that influence their continued connection and pro-environmental behaviour. Research from Singapore has shown a person’s connection to nature is influenced by family values regarding nature, social norms of family and friends as well as personal experiences of nature (Oh et al. 2021). Research on Family Nature Clubs, community-based organizations that regularly bring families together to enjoy nature together, have also shown that the significant life experiences that influence environmental values and behaviours are based on a triad of experiences that include free play and exploration in nature in childhood or youth, influential role models who communicate nature’s value, and opportunities to learn how to take action on nature’s behalf (D’Amore and Chawla 2020). Such research shows the influence that parents and family can have on the development of nature connection (Passmore et al. 2021). Some people have also mentioned that they are developing meaningful nature experience from spending time in nature with their children or grandchildren, and this provides new opportunities for adults to connect with nature as well.

### Understanding the variability of Australia cities

Australia is one of the most urbanised nations in the world, with most people living in towns and cities, and along the coast (Australian Institute of Health and Welfare 2024). Thus, the Inner Regional set of survey participants are still near major urban centres in coastal regional cities, while Outer Regional and Very Remote/Remote communities are often located further inland with the population becoming sparser the further inland people are. Very Remote/Remote communities can be located in quite isolated parts of Australia, far away from transportation nodes, health services, and communication opportunities like internet and post offices, leading to inequalities in access and reduced assistance for vulnerable populations (Taylor et al. 2017).

However, like many other countries, Australia’s major cities also tend to have a different demographic than regional areas. The urban population tends to be younger and more diverse than in regional areas; capital cities have historically attracted more young people from overseas and regional areas who move to the cities to pursue educational and job opportunities (Australian Institute of Health and Welfare 2024; ABS July 2021–June 2026). In the opposite direction, people of retirement age sometimes move to regional areas for a lifestyle change, resulting in compounding the older demographics in regional areas (Australian Institute of Health and Welfare 2024; ABS July 2021–June 2026). These trends likely apply more to Outer Regional areas than to Remote/Very Remote, especially considering the lack of services found in Remote/Very Remote areas. Previous research has shown that older Australians, people without children at home and those with more time availability tend to have higher levels of nature connection; and thus, the demographics of these more remote geographies may help explain the higher levels of nature connection that in seen in the present research (Dean et al. 2018; Sollis et al. 2025).

It is also important to note that major cities in Australia are quite varied in their size, infrastructure, and history of development. Major cities across states and territories are highly variable, with Sydney and Melbourne combined representing almost half the population of Australia (Australian Institute of Health and Welfare 2024). Many of the other major cities are smaller and growing, but represent slower development with regional-type aspects to planning and infrastructure as well as social networks and economic productivity (Azpitarte et al. 2021; Ho et al. 2024). Thus, within the Major Cities group, there is still significant variation in the environment surrounding these individuals; this type of variation would be reflected within all of the classifications used in this paper suggesting that place-based strategies for nature connection would be most effective.

Previous studies have found some inconsistencies regarding relationships between pro-environmental behaviour and regional context and across the urban–rural gradient. Some studies have found that people living in more rural areas to have greater pro-environmental behaviors, often assumed because they are surrounded by nature and therefore more in touch with nature (Duron-Ramos et al. 2020; Sierra-Barón et al. 2023). Other studies have found that people living in cities with less nature immediately available exhibit more pro-environmental perhaps because there are more possible ways to be sustainability conscious, including exposure to different campaigns, access to recycling systems, more agency to be involved in this types of activity (Berenguer et al. 2005; Gifford and Nilsson 2014). Additionally, it is important to consider that some people in rural contexts have lifestyles and jobs that are more oriented to unsustainable or nature extractive type industries, which is the case within Australia and discussed above (Brueckner et al. 2013).

### Limitations and future directions

It should be emphasised that as this study was conducted exclusively in Australia, and the unique qualities of geographical population distribution may impede the extent to which the findings can be transferrable to other regions of the world; however, many areas of the world have gone through a similar urban transition and thus still have a large gradient between remote, to regional, to urban areas of different sizes. Thus, although the pattern of population size and distribution is unique to Australia, variations of the urbanisation gradient and pattern of distribution are universal across countries.

A limitation of this study may be that the survey was only distributed electronically and in one language (English). Because of this reason, the survey may not have been accessible to Aboriginal Australians or culturally diverse members of the population who do not read English, as may be supported by the data in Table 1 showing that the percentage of survey participants for “Identifies as Indigenous” and “Linguistically diverse” was lower than the national benchmark figure. For First Nations people in Australia, it is estimated that 40 per cent of adults have minimal English literacy and this figure can rise as high as 70 per cent in remote communities, such as those in the Northern Territory ((Australian Government 2023). In remote areas of Australia, there are also more limitations on services, which includes electricity, internet, and technology and the infrastructure to support these systems. Because of these limitations to communications services, it was more difficult to survey remote communities with the depth and breadth of the other regions, and especially with-in vulnerable populations that may not be digitally literate {Australian Digital Inclusion Index, 2023 #458).

However, the numbers of survey participants obtained from this research are representative of the benchmark population percentages for each remoteness region as classified by Australia’s Bureau of Statistics and 2021 Census information (Table 1). Thus, for the Australian context, the results of the study have relevance toward decision-making and national strategies toward improving nature connection, personal wellbeing, and pro-environmental behaviour of communities across the different remoteness categories and for the nation as a whole. First, we see that the Outer Regional category had survey participants that reported the highest nature connection, personal wellbeing, and pro-environmental behaviour scores. Such a pattern indicated that there may be greater availability of nature access or engagement than the Major Cities or Inner Regional areas, there is sufficient services and infrastructure to allow for high wellbeing, and there are opportunities and support for environmental values to be translated into specific behaviours. Understanding the qualities and mix of benefits from this type of regional development could be very useful for designing and developing communities in the other remoteness categories. Future research should focus on understanding these different qualities and trying to scale or transfer them to other remoteness categories.

Additionally, the results highlight the value of childhood experiences in nature and the values of the experiences for these individuals as they recall these experiences as adults. When survey participants were asked about their most meaningful nature experience, many participants recalled immersive experiences in nature as children, and especially recalled how these experiences were shared with friends and family who took the time and interest to teach how to appreciate and spend time in nature. Such acknowledgment of the significance of these experiences may be so memorable not only because of the nature experience, but also because of the recognition of the strong social and emotional connection they had with the people who were in the experience with them. This interaction is complex and further research should aim to better understand the interaction of the values that are developed through such experiences.

## Conclusions

Understanding experiences with nature across the rural to urban gradient is important for understanding the extinction of experience, and the changing ways that individuals view and value nature. This study shows that Outer Regional and Very Remote/Remote areas of Australia have higher levels of nature connection than in Inner Regional and Major Cities areas, confirming that urbanisation can have an impact on nature connection. Considering 78% of Australians live in Major Cities or Inner Regional areas, it is particularly concerning that these two categories have lower pro-environmental behaviours than Outer Regional (where only 18% of the population lives). Nevertheless, this study shows that the wellbeing and PEB relationships are non-linear, with individuals in Outer Regional areas having the highest level of wellbeing and PEBs, while Very Remote/Remote areas of Australia matched Inner Regional and Major City populations for these two variables. This finding reflects the diverse factors influencing these metrics, and the complex context for nature connectedness in Australia.

A hopeful finding from this study is that meaningful childhood experiences were shown to be impactful regardless of where people lived as adults, but as the population becomes more urbanised, opportunities to have these immersive experiences must be available to urban children. These formative experiences shape our values and actions around nature and willingness to visit and take care of it. For some, these experiences can generate regret and a sense of loss for the world as it might once have been. However, solutions for preserving nature cannot be based on notions of a simple return to the past and a dismantling of current urbanisation. Understanding how and why outer regional areas of Australia preserve both nature connection and high levels of well-being and pro-environmental behaviour can help us understand what levers are available to improve life in major cities.

## Supplementary Information

Below is the link to the electronic supplementary material.

Supplementary file1 (PDF 597 KB)

## Footnotes

1
https://www.abs.gov.au/statistics/standards/australian-statistical-geography-standard-asgs-edition-3/jul2021-jun2026/remoteness-structure/remoteness-areas.
