# Supplementary material for: Nature connection, wellbeing and pro-environmental behaviour across an urban gradient: Understanding the regional sweet spot
Source: Ambio. 2025 Aug 31;55(2):327–43. doi: 10.1007/s13280-025-02229-2 (PMC12779833; doi:10.1007/s13280-025-02229-2)
Supplement: Supplementary file 1 — Supplementary file1 (PDF 597 KB) [file 13280_2025_2229_MOESM1_ESM.pdf]

Supplementary Information

Nature connection, wellbeing and pro-environmental behaviour across an urban gradient: Understanding the regional sweet spot

Brenda B. Lin, Kate Sollis, Emily J. Flies, and Pauline Marsh

Table S1. Table of the results of the Tukey Honest Significant Difference tests for nature connection (a), personal wellbeing (b), and pro-environmental behaviour (c). Signif. codes: 0 '***' 0.001 '**' 0.01 '*' 0.05 '.' 0.1 ' ' 1 denote significant differences between the two categories. Lower Bound/Upper Bound: the lower and upper-end points of the confidence interval at 95 percent; P-value adjusted: p-value after multiple comparisons adjustment.

(a) Nature Connection

Group comparisons	Difference	Lower Bound	Upper Bound	P-value adjusted
Outer Regional-Major City	0.332	0.204	0.460	0.000***
Inner Regional-Major City	0.076	-0.038	0.191	0.314
Very Remote/Remote-Major City	0.322	0.073	0.571	0.005**
Inner Regional-Outer Regional	-0.256	-0.401	-0.110	0.000***
Very Remote/Remote-Outer Regional	-0.010	-0.275	0.254	0.999
Very Remote/Remote-Inner Regional	0.245	-0.013	0.504	0.070.

(b) Personal Wellbeing

Group comparisons	Difference	Lower Bound	Upper Bound	P value adjusted
Outer Regional-Major City	4.019	0.844	7.194	0.006**
Inner Regional-Major City	-0.259	-3.101	2.584	0.996
Very Remote/Remote-Major City	-1.411	-7.898	0.656	0.936
Inner Regional-Outer Regional	-4.277	-7.898	-0.656	0.012*
Very Remote/Remote-Outer Regional	-5.430	-12.014	1.154	0.147
Very Remote/Remote-Inner Regional	-1.153	-7.583	5.277	0.968

(c) Pro-environmental Behaviour

Group comparisons	Difference	Lower Bound	Upper Bound	P value adjusted
Outer Regional-Major City	0.102	0.011	0.194	0.021*
Inner Regional-Major City	0.008	-0.074	0.089	0.995
Very Remote/Remote-Major City	0.003	-0.175	0.180	1.000
Inner Regional-Outer Regional	-0.095	-0.199	0.009	0.090.
Very Remote/Remote-Outer Regional	-0.100	-0.288	0.089	0.524
Very Remote/Remote-Inner Regional	-0.005	-0.189	0.179	1.000

Table S2. Correlations and significance value for pairwise comparisons between CN12 (nature connection scale), PWI (Personal Wellbeing Index), and PEB (Pro-environmental behaviour). Separate analyses were conducted for each of these factors within the main manuscript because these factors are correlated, but they provide different information regarding the individual.

**Remote/Very Remote**	Correlation co-efficient (r)	p-value
CN12-PEB	0.2416	<0.000
PEB-PWI	0.0202	<0.000
CN12-PWI	0.0176	<0.000

**Outer Regional**	Correlation co-efficient (r)	p-value
CN12-PEB	0.2416	<0.000
PEB-PWI	0.0202	<0.000
CN12-PWI	0.0176	<0.000

**Inner Regional**	Correlation co-efficient (r)	p-value
CN12-PEB	0.2416	<0.000
PEB-PWI	0.0202	<0.000
CN12-PWI	0.0176	<0.000

**Major Cities**	Correlation co-efficient (r)	p-value
CN12-PEB	0.2416	<0.000
PEB-PWI	0.0202	<0.000
CN12-PWI	0.0176	<0.000

Figure S1. Types of everyday interactions with nature that are coded based on open-text responses to the question "What kind of interactions do you have with nature in your everyday life? Please describe in your own words. (optional)". The responses have been coded and then counted for frequency within each remoteness category. The total number counted was then scaled to the total respondent number to gain an understanding of the percentage of respondents who responded with the coded subtheme. The information is displayed by remoteness category.

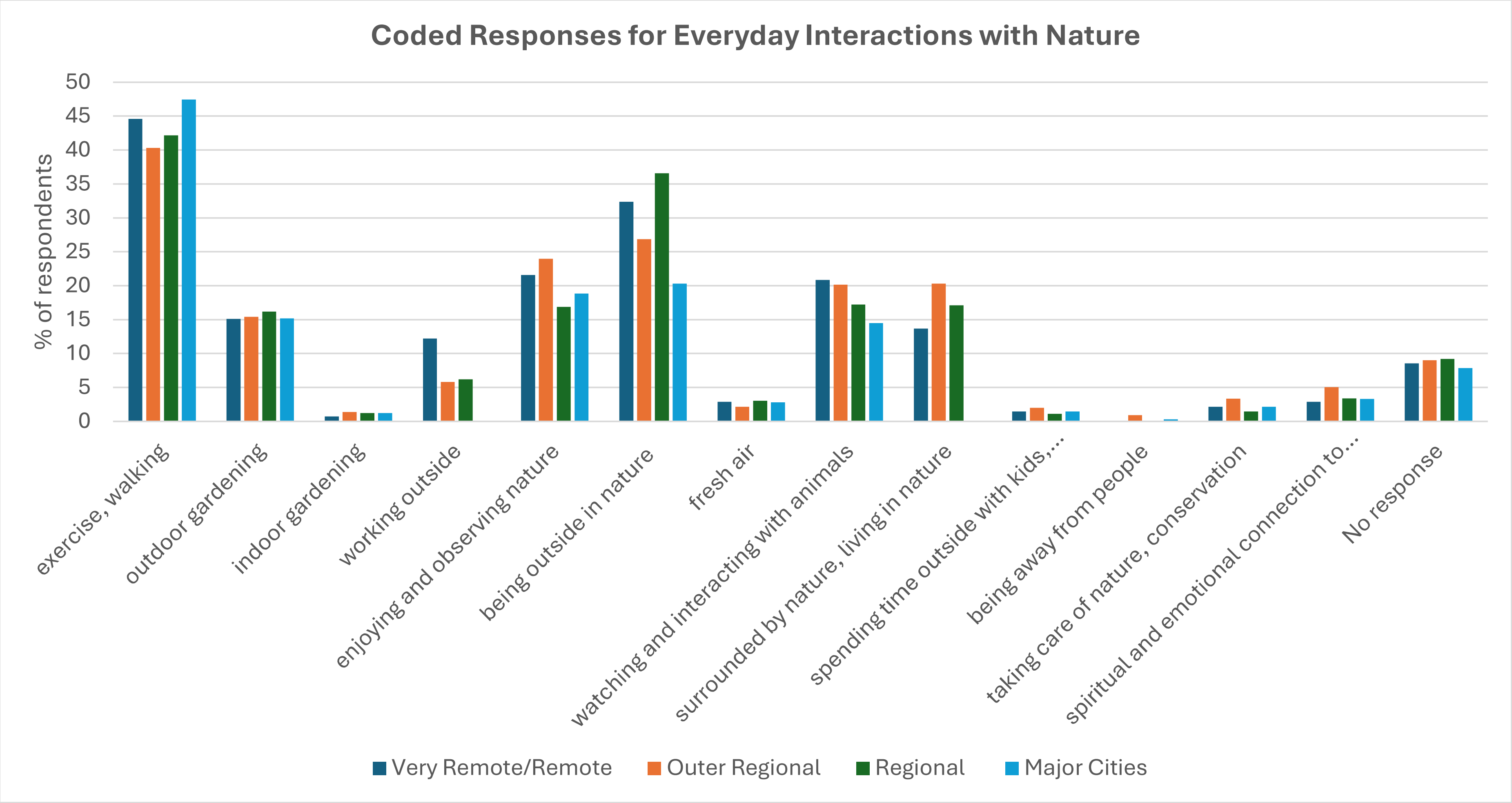

Figure S2. Types of meaningful experiences with nature that are coded based on open-text responses to the question "Think of a meaningful experience you have had that shaped the way you think about 'nature'? Please describe it in 1-3 sentences in your own words. (optional)". The responses have been coded and then counted for frequency within each remoteness category. The total number counted was then scaled to the total respondent number to gain an understanding of the percentage of respondents who responded with the coded subtheme. The information is displayed by remoteness category.

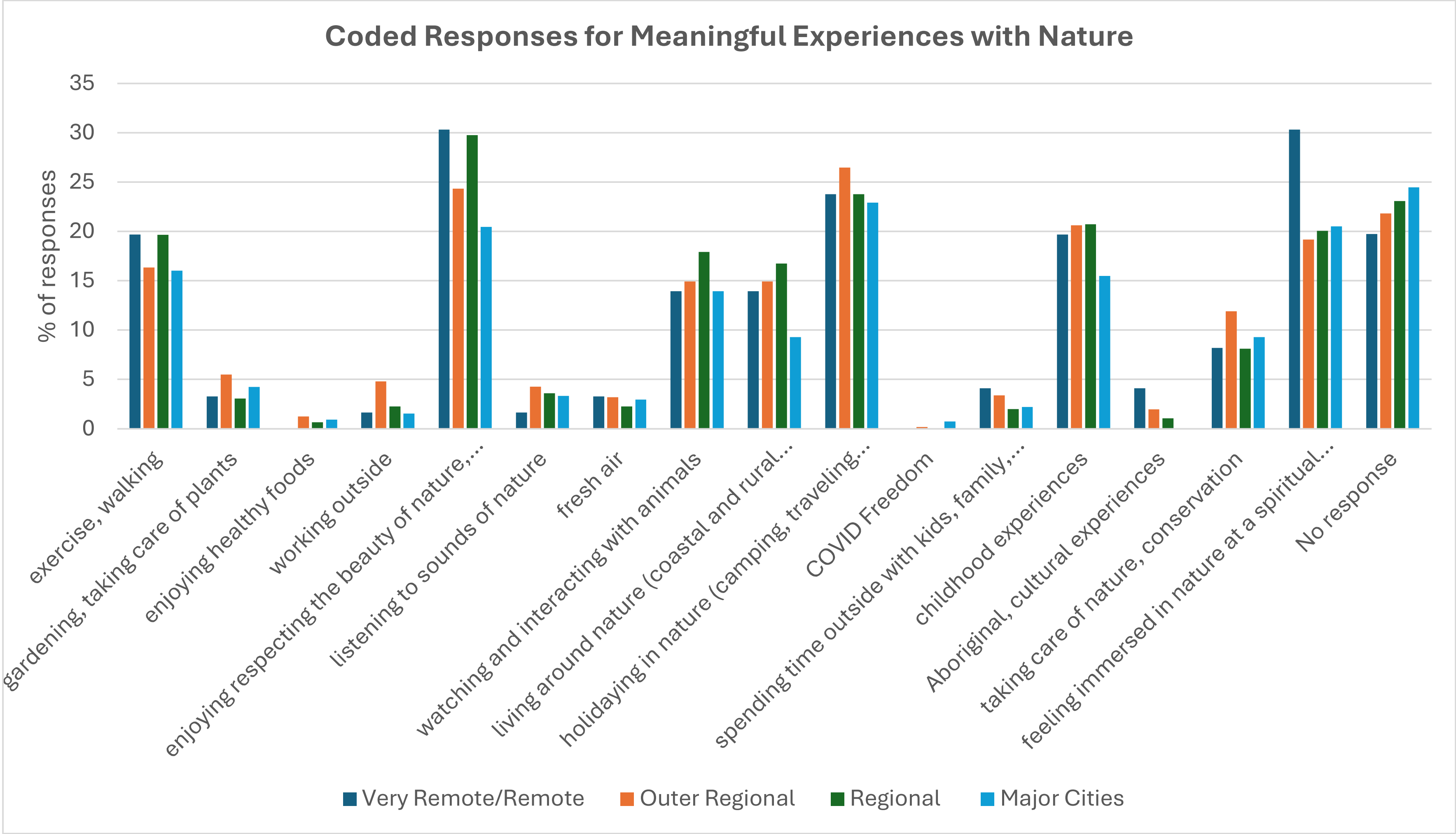

Table S3. Exemplary quotes by subtheme across the four groups for Q1: Everyday Interactions with Nature.

	Very Remote/Remote	Outer Regional	Inner Regional	Major Cities
**COMMONALITIES**				
**Exercising, walking**	• "I am outdoors every day in nature - running, biking, gardening, walking; even just hanging out washing, listening to the birds, looking at the sky; swimming in the ocean; being 'in it'" (ID 3857)	• "I try to get as much outdoor and nature into my day and it's much easier living in a regional town in a part of town with many parks and street trees. I often try to get sunlight and spend time walking." (ID 3392)	• "Walk on the beach regularly - without headphones!" (ID 3234)<br>• "Daily dog walks on 100-acre property" (ID 4157)	• "I walk/run in my neighbourhood" (ID 268)<br>• "I do a lot of walking and some running. I am outdoors a lot." (ID 5243)
**Enjoying and observing nature**	• "I am walking outside everyday and I am having my feet planted on the ground and be breathing the air and be looking at the sun, the moon, and up at the sky" (ID 2080)<br>• "I'm always outside sitting on the ground, sitting at the trees, admiring the sky and the country" (ID 7139)	• "We have a dam on our property and see the ducks and their babies. see the cows next door and our beautiful birds that come into our garden. forest just up the road and beach 10 minutes away. We are very lucky." (ID 760)<br>• "I live on 20 hectares - every day I enjoy the changing light - the seasonal changes and just getting outdoors without the noise and hassle of cities" (ID 2847)	• "I sometimes sit out in the backyard and watch the birds in the trees" (ID 2554)<br>• "I have a lovely garden to sit and look out on or work in if weather permits. I enjoy watching and listening to all the birdlife in the area." (ID 2689)	• "Go down to a secluded park and watch the birds etc. out on the water in the estuary" (ID 28)<br>• "walking in the park by the lake and in the bush, listening to birds sing, feeling wind, watering plants in the garden and indoors" (ID 163)
**Gardening outdoors**	• "I love to garden, watering my grass and plants, enjoy having plants inside my home, and I go for bush walks on my days off." (ID 2871)	• "Gardening daily. I get intimate with the garden when I do so." (ID 1922)<br>• "Gardening and listening to the birds, enjoying the beautiful flowers" (ID 2562)	• "working in the garden, planting vegetables" (ID 1482)<br>• "I love to do the gardening when I'm at home." (ID 2615)	• "Gardening to look after vegetable growing and flower growing." (ID 180)
**DIFFERENCES**				
**Watching and interacting with animals**	• "Have a bird bath that we fill daily and love listening to the birds, have an inside only cat so our garden is filled with lizards, Tata lizards, gilbert dragons, a monitor lizard, blue tongues" (ID 5660)	• "Looking after animals and living in a beautiful rural area surrounded by forestry." (ID 1741)	• "Seeing native wildlife on the urban fringe, respecting nature" (ID 370)	• "I watch the birds in the backyard from my living room" (ID 2558)
**Surrounded by nature, living in nature**	• "I live and work in a remote bushy area" (ID 3372)<br>• "I live in a remote town so we have a lot of wildlife around our hotel property and are surrounded by Australian bush" (ID 8110)	• "I live rural, surrounded by land and bush, plenty of gum trees and birds, during the night we get many wallabies grazing around us, plus gliders, possums and fruit bats." (ID 386)	• "Being in a small rural town we have beaches everywhere" (ID 145)<br>• "I live on 5 acres in semi-rural area, so every day I feel connected to nature. I go outside each day to be with my horses, to look after my kangaroos." (ID 815)	
**Being outside in nature**	• "Everything. I spend 90% of my time outside Watering the garden, looking at the trees" (ID 5202)• "I currently am working in a small community in Central Australia in the desert so I am inside nature every day." (ID 7541)	• "We enjoy Sunshine and rain and wind. Often outside with no shoes on when the weather isn't too inclement" (ID 848)	• "I live in an urban, yet bushy environment with abundant wildlife in my backyard" (ID 1253)	• "In my everyday life, I try to spend as much time as I can outside. I enjoy going for walks in the park and hiking in the mountains. I also love to garden and grow my own fruits and vegetables. It's always a great

				feeling to be able to enjoy the fruits of my labour!" (ID 6842)
**Working outside**	• "I work adjacent to a national park nominated for World Heritage, I work in this national park at times collecting data, admiring the beauty" (ID 3755)<br>• "I'm a ranger, so I have a lot of interaction with wildlife and nature such as plants, waterfalls etc." (ID 5210)	• "I spend part of my job outside in nature. I take some of my clients for walks, picnics, to zoos to see animals." (ID 1703)<br>• "I work in agriculture as a fencing contractor and enjoy just being in and around the bush and farmland and enjoy the quiet of it all." (ID 2972)	• "Work in a national park" (ID 5887)<br>• "I work outside so a lot" (ID 6030)<br>• "Farm work" (ID 5794)	
**Spiritual-emotional connections**	• "I feel the need to be connected to the 'outside'. If I am in a room in my residence or office I MUST have a door open and I must be able to see OUTSIDE no matter how hot it gets here at times… I NEED Fresh Air." (ID 4617)<br>• "Love and healing" (ID 5882)	• "I try to walk around outside at least once a day with bare feet to feel reconnected with "the earth" (ID 2620)<br>• "I live very much connected to Mother Nature" (ID 3333)	• "I connect with the plants, birds and bees in my garden." (ID 3041)<br>• "Everything. From the environment, animals (birds in particular), the plants, water . Everything. I feel connected to it all the time" (ID 4944)	
**Conservation**	• "Making sure my rubbish is sorted, using electricity carefully, planting native trees mainly," (ID 656)	• "I try to live as sustainably as possible and leave the smallest possible footprint" (ID 2748)	• "Am currently involve in bush regeneration and am always planting native rainforest trees" (ID 61)	• "Volunteer Bushcarer at 3 sites, Bushwalking club outings weekly" (ID 9128)
**Spending time with children outdoors**	• "I enjoy being outside, walking, taking the children to the park and flowers" (ID 5387)	• "I show tree birds moon sky to my kids and tell story about them" (ID 6310)	• "Walking outside and playing with my children"	• "Showing children at the daycare centre I work with how to connect and create with nature." (ID 3531)
**Looking outside the window to nature**		• "Water views, beach walks, driving through bush & hills. Watching native animals & birds in the yard" (ID 1130)	• "Drive through natural environment" (ID 732)<br>• "The view from my home. the drive to work and in my garden" (ID 2943)	• "Looking at views from third floor of building at work" (ID 3881)<br>• "Almost nothing, just driving thru" (ID 2422)
**Cultural Connection**	• "Being outdoors with my family, friends and pets. And going adventuring with them to find connectedness in my country." (ID 6503)	• "Connecting with Country" (ID 4260)		
**Minimal or limited time outside**	• "Not too much. Limited only by my laziness." (ID 3067)	• "Not enough. I work in an office." (ID 3811)	• "Living in the CBD (central business district) interactions with nature now take conscious effort to "get out" (ID 2571)<br>• "Nothing because of the choice of living in a city" (ID 5078)	• "Not much. There is a creek near me but sometimes I feel unsafe walking along there." (ID 361)<br>• "Minimal, I wish it was more, but most time these days is spent working and saving money." (ID 6194)
**Adversarial feelings**	-			• "None I know of but got flaming pidgins roosting on the roof and making a hell of a mess" (ID 570)<br>• "As little as possible" (ID 1852)

Table S4. Exemplary quotes for the various subtheme across groups for Q2: Meaningful Experiences with Nature.

	Very Remote/Remote	Outer Regional	Inner Regional	Major Cities
**COMMONALITIES**				
**Exercising, walking**	• "Great walk in the bush where there is no cars." (ID 3360)<br>• "A walk to a lookout last weekend, reminded me how lucky I am to live here and not in a city" (ID 3624)	• "Walking on the beach at dusk or climbing a mountain." (ID 2036)<br>• "Bush walking and camping, cross country skiing, walking along a beach" (ID 2954)	• "Walking and fly fishing in the western lakes world heritage area." (ID 448)<br>• "Walking through Scandinavia and experiencing nature at a different level and physically feeling a change" (ID 1662)	• "Recent walking trip to Tasmania." (ID 55)<br>• "Beach walks on holidays and admiring Australia and overseas countries" (ID 94)
**Holidaying in nature**	• "Camping as a young adult and on the third day realised I had forgotten the date (which was really hard to do because I worked in a library) and I just felt so relaxed for the first time." (ID 3038)	• "I travel to see nature at its best wherever and whenever I can .I like to photograph and study the natural environment for my own enjoyment." (3162)	• "Wintering in Antarctica is an experience very few people have the privilege to enjoy. The beauty and the ferociousness of nature coexist." (ID 2984)	• "Climbing Kilimanjaro, trekking in the Himalayas, and country walking in Europe all have given me a sense of calmness and awe at the world in which we live." (ID 2920)
**Childhood experiences**	• "Since I was a child I have gone bush with my parents then with my own children and now with my children." (ID 3706)<br>• "Growing up hunting and fishing and being taught about nature by my father and uncles." (ID 4750)	• "Fishing at the local spot with my family as a child was my favourite thing to do." (ID 2620)<br>• "Camping in the central Australian desert as a child, the vastness and isolation was peaceful and serene then" (ID 1648)	• "I grew up on a property and learnt many things from my grandfather." (ID 3982)<br>• "Taking holidays as a child with my family to nature settings. These experiences culminated into a deep love of nature-based holidays and escaping the city." (ID 4080)	• "When I was about 7 I went to a holiday activity in the state forest. From then on I knew I loved nature. as a child my mother looked after recovering wildlife and we had a pool full of tadpoles that I often swam with. Nowadays I go to nature to reset my mind." (ID 792)
**Respecting the beauty of nature**	• "Serenity and beauty of nature is everywhere and always has been. We need to take the time to be aware of its awesome power." (ID 3386)	• Experiencing life on the road exposed to the elements living simply in a swag you quickly appreciate the natural beauty this country and the world has to offer and how much of a shame it is we so readily destroy it." (ID 848)	• "Went to the Daintree in Qld and realised how old nature is and the sheer strength and beauty of nature. Realising the forest was over 1 million years old made me feel insignificant and appreciative of nature." (ID 3267)	• "A visit to the Redwood trees in San Francisco, nature is far more magnificent that we appreciate" (ID 4652)
**DIFFERENCES**				
**Watching and interacting with animals**	• "Experiencing a number of encounters with birds and animals that have occurred while out and about, both in the garden and while travelling." (ID 2670)<br>• "Surfing and encounters with marine animals" (ID 7821)	• "Watching kangaroos eat the grass, hearing the birds singing, hearing the wind whistling through the trees." (ID 3221)<br>• "Walking on forest paths. Sighting Cassowaries, Monitor lizards" (ID 3116)	• "Having a family of ring tail possums living in our garden" (ID 2064)<br>• "I cuddled a wombat & fell in love" (ID 2094)	• "I was befriended by a local magpie who brought her babies for a visit." (ID 4273)
**Living around nature**	• "Growing up on a small farm in the countryside with many animals was a memorable childhood. Now live in remote WA where we go camping and fishing." (ID 4381)<br>• "During the wet season it floods regularly near where I live. It became a	• "Being a farming/grazing family, we lived by what nature delt us - plenty in good seasons, less birds and animals or hungry animals during drought." (ID 3081)<br>• "I see beautiful natural undisturbed areas on a daily basis. I live in a rural	• "I moved to a rural property after living in apartments for 25 years." (ID 1664)<br>• "Not being in a city. I have nature on my doorstep." (ID 2035)	

	bird sanctuary for the next six months." (ID 4441)	town which has many areas to explore and enjoy." (ID 3648)		
**Feeling immersed in nature at a spiritual level**	• "Travelling in the outback of Australia, experiencing the expanse of it, made me want to stop and just be in the moment; I try every day to do this now." (ID 3857)	• "Walking around Uluru, absolute beauty and spiritual place that connects you to nature. Also, the wild storms of the wet season in North Australia make you feel alive." (ID 2570)	• "Swimming in ocean pools when I lived in Sydney felt protected but connected to the water. Walking in the rainforest near where I live filled me with wonder." (ID 4181, Regional)	
**Connection to nature**	• "I feel more connected, relaxed and myself when I can be outdoors and in nature, I especially feel this when I'm completely away from technology." (ID 2871)	• "Enjoying a breathing and relaxation session after I attended an exercise class held on the grass in the shade of some beautiful old trees, looking out over the aqua coloured water of a bay." (ID 3637)	• "Walking through a natural park and being able to feel refreshed by the tranquillity, serenity and natural beauty of creation." (ID 732)	
**Religious connotations**			• "Just know that it was created by God for us to enjoy." (ID 832)<br>• "Completing a bushwalk in Tasmania made me think that God created an awesome world" (ID 3257)	• "Being closer to God and seeing His hand in all creation" (ID 1375)<br>• "Faith in the concept of 'creation' (as described in the Torah and Bible)." (ID 5943)
**Aboriginal, cultural experiences**	• "Meeting with Aboriginal Elders in 1978 during the Employment Campaign to eradicate the Aedes aegypti Mosquito." (ID 656)<br>• "The more work I do with Indigenous communities. Getting to understand the connection with land - touch - the importance of looking after it." (ID 3972)	• "Interacting with Indigenous peoples in Africa. Seeing how people live off the land and how they walk hand in hand with nature." (ID 3317)<br>• "Living at the foot of Gulaga and learning about the local Indigenous connection to the mountain." (ID 5208)	• "Being Aboriginal I feel like I have a huge connection to nature" (ID 1596)<br>• "Just the way I was bought up. Being connected to everything and everything to you. Especially on my own country (woka). Yorta Yorta Woka!" (ID 4984)	• "When visiting Uluru it was easy to understand the significance of the area." (ID 7083)
**Covid-19**				• "Covid19, made me more appreciate nature" (ID 60)<br>• "Being indoors because of COVID made me appreciate nature more" (ID 823)
**Minimal or limited meaningful experiences**		• "Always loved nature nothing in particular comes to mind" (ID 4550)	• "a few camp trips were good but nothing really of note" (ID 7543)	• "I have had no "meaningful" experiences with nature. It's not an entity, a consciousness - it's just plants and animals." (ID 68)<br>• "None? Nature just exists and is nature. I don't have any particularly meaningful experiences in it." (ID 839)
